# Combined saline and vildagliptin induced M2 macrophage polarization in hepatic injury induced by acute kidney injury

**DOI:** 10.7717/peerj.14724

**Published:** 2023-02-13

**Authors:** Shaimaa N. Amin, Hader I. Sakr, Walaa B. El Gazzar, Sherif A. Shaltout, Hazem S. Ghaith, Dalia A. Elberry

**Affiliations:** 1 Department of Anatomy, Physiology, and Biochemistry, Faculty of Medicine, The Hashemite University, Zarqa, Jordan; 2 Department of Medical Physiology, Faculty of Medicine, Cairo University, Cairo, Egypt; 3 Department of Medical Physiology, Medicine Program, Batterjee Medical College, Jeddah, Saudi Arabia; 4 Department of Medical Biochemistry and Molecular Biology, Faculty of Medicine, Benha University, Benha, Egypt; 5 Department of Pharmacology, Public health, and Clinical Skills, Faculty of Medicine, The Hashemite University, Zarqa, Jordan; 6 Department of Pharmacology, Faculty of Medicine, Benha University, Benha, Egypt; 7 Faculty of Medicine, Al-Azhar University, Cairo, Egypt

**Keywords:** Dipeptidyl peptidase 4 inhibitor, Vildagliptin, Liver, Acute kidney injury, MAPK/AP-1

## Abstract

Acute kidney injury (AKI) is a prevalent medical condition accompanied by mutual affection of other organs, including the liver resulting in complicated multiorgan malfunction. Macrophages play a vital role during tissue injury and healing; they are categorized into “classically activated macrophages” (M1) and “alternatively activated macrophages” (M2). The present study investigated and compared the conventional fluid therapy *vs* Dipeptidyl peptidase 4 inhibitor (DPP-4i) vildagliptin on the liver injury induced by AKI and evaluated the possible molecular mechanisms. Thirty rats comprised five groups (*n* = 6 rats/group): control, AKI, AKI+saline (received 1.5 mL of normal saline subcutaneous injection), AKI+vildagliptin (treated with oral vildagliptin 10 mg/kg), AKI+saline+vildagliptin. AKI was induced by intramuscular (i.m) injection of 50% glycerol (5 ml/kg). At the end of the work, we collected serum and liver samples for measurements of serum creatinine, blood urea nitrogen (BUN), alanine aminotransferase (ALT), aspartate aminotransferase (AST), tumor necrotic factor-α (TNF-α), and interleukin-10 (IL-10). Liver samples were processed for assessment of inducible nitric oxide synthase (iNOS) as a marker for M1, arginase 1 (Arg-1) as an M2 marker, c-fos, c-Jun, mitogen-activated protein kinase (MAPK), activator protein 1 (AP-1), and high-mobility-group-box1 (HMGB1) protein. The difference was insignificant regarding the relative expression of AP-1, c-Jun, c-fos, MAPK, and HMGB between the AKI+saline group and the AKI+Vildagliptin group. The difference between the same two groups concerning the hepatic content of the M1 marker (iNOS) and the M2 marker Arg-1 was insignificant. However, combined therapy produced more pronounced changes in these markers, as the difference in their relative expression between the AKI+saline+Vildagliptin group and both the AKI+saline group and the AKI+Vildagliptin group was significant. Accordingly, we suggest that the combined saline and vildagliptin hepatoprotective effect involves the downregulation of the MAPK/AP-1 signaling pathway.

## Introduction

Acute kidney injury (AKI) is a relevant debility among critical cases that requires intensive care admission with multiple organ cross-signaling, leading to malfunction and failure (Rewa & Bagshaw, 2014). Renohepatic cross talk is a reported outcome because of direct complications of AKI or systemic and cytokine-dependent mechanisms (Bonavia & Stiles, 2021).

Macrophages represent a complex group of immune cells crucial for physiological homeostasis and responses to pathological insults. Relying on the surrounding microenvironmental stimuli, macrophages differentiate distinctly into two phenotypes in a process called macrophage polarization (Hume, 2008). Polarized macrophages have two main categories: classically activated macrophages (M1), which are capable of proinflammatory responses, and alternatively activated macrophages (M2) which promote tissue remodeling, cell proliferation, and immune regulation (Martinez et al., 2008). Significant studies revealed the contribution of macrophage subtypes and heterogenicity in AKI (Li et al., 2021; Wen et al., 2021) and acute liver injury (Sun et al., 2017; Bai et al., 2021).

M1 macrophages, which are inducible by either interferon-γ (IFN-γ) alone or in conjugation with other cytokines or microbial stimuli, are marked by high production of reactive oxygen species (ROS), proinflammatory cytokines, and nitric oxide (NO) (Verreck et al., 2004). Conversely, M2 macrophage polarization upregulation depends on multiple stimulatory factors, including immune complexes, glucocorticoids, and cytokines (Martinez, Helming & Gordon, 2009).

One of the critical aspects of macrophage polarization is the alternation in the expression of cell surface markers like different clusters of differentiation (CDs) and other markers. M1 macrophages are marked by inducible nitric oxide synthase (iNOS), while arginase 1 (Arg-1) is the M2 macrophage marker (Van Dyken & Locksley, 2013).

Dipeptidyl peptidase 4 inhibitors (DPP-4i), the incretin-based anti-diabetic medications, are abundant in endothelial and epithelial tissues. Inhibiting DPP-4 signaling suppresses the high glucose-induced generation of ROS and has antiapoptotic, anti-inflammatory, and antioxidative effects (Wiciński et al., 2020). Besides, DPP-4i showed immunomodulatory actions on several immune cells, including the function of macrophages and the regulation of M1/M2 polarization (Shao et al., 2020). It is worth noting that the DPP-4i alleviates hepatic damage in steatosis and non-alcoholic fatty liver disease (NAFLD) and diethylnitrosamine (DENA)-induced liver cancer (Jung et al., 2014; Jiang et al., 2018); however, the effect on liver injury complicating AKI is not well reported.

The morbidity and mortality rates with hospital admission are real challenges in health care practice, and it needs to be reduced for AKI cases complicated by organ failures like hepatic failure. Conventional fluid therapy has beneficial effects for these patients; however, an unsatisfactory outcome still favors the finding of other management plans. Besides, the mechanism of crosstalk between the liver and kidney in AKI needs further clarification. The current work aimed to study the effect of DPP-4i vildagliptin on the hepatic function and macrophage polarization in an AKI animal model and investigate the possible molecular mechanisms, and the tested hypothesis was that vildagliptin has a hepatoprotective effect.

## Materials and Methods

The Institutional Review Board of Hashemite University approved the experimental steps, animal handling, sampling, and euthanasia (approval number: 5/7/2020/2021). The handling of animals followed the “Care and Use of Laboratory Animals guide” (National Research Council (US) Committee for the Update of the Guide for the Care and Use of Laboratory Animals, 2011).

### Experimental animals and groups

The animal model for this study consisted of 30 Wistar albino adult male rats weighing between 150 and 200 grams, aged 70–75 days. Animals were housed in cages as three rats per cage to avoid isolation stress that might produce bias in our results. Rats were acclimatized to normal environmental conditions for 1 week regarding dark/light cycle, temperature (22 ± 5 °C), and relative humidity (45 ± 5%), with unrestricted access to water and laboratory food. The rats constituted five groups, and the sample size was based on similar studies using the minimal possible number of rats (6 rats/group):

*Group I (Control group):* used as the normal values to which measurements in other groups were referred.

In the other groups, AKI was induced by intramuscular (im) injection of 50% glycerol (5 ml/kg) (Homsi et al., 2002). The dose was divided into two halves; each was injected into one hind limb muscle. The rats were water-deprived 24 h before glycerol injection. After injection, conscious rats were placed in cages with unrestricted food and tap water access. They were divided at random to form four groups as follows:

*Group II (AKI):* injected with glycerol without further treatment but received 3 ml distilled water by oral gavage (vehicle).

*Group III (AKI+ saline):* received 1.5 ml of normal saline subcutaneous injection with glycerol injection and then every 12 h for 72 h (Miyaji et al., 2003).

*Group IV (AKI + vildagliptin):* Treatment with daily oral vildagliptin 10 mg/kg (Galvus; Novartis Pharma Stein AG, Stein, Switzerland) (Sherif, Alshaalan & Al-Shaalan, 2020) started 1 h after glycerol injection and continued with the same dose every 24 h for 72 h.

*Group V (AKI + saline+ vildagliptin):* received saline and vildagliptin as previously mentioned in groups III and IV.

Randomization was used to allocate experimental units to control.

Treatment was given at the same time of the day, and cages and rats were numbered to minimize potential confounders, such as the order of treatments and measurements or animal/cage location and treatment groups.

We euthanized the animals by cervical dislocation (3 h after the last dose), dissected the abdomen, collected blood samples from the aorta, and extracted the liver when the experimental steps terminated. Following blood and liver samples collection, serum creatinine, blood urea nitrogen (BUN), alanine aminotransferase (ALT), aspartate aminotransferase (AST), tumor necrotic factor-α (TNF-α), and interleukin-10 (IL-10) were measured. Liver samples harvested for assessment of iNOS, Arg-1, c-fos, c-Jun, mitogen-activated protein kinase (MAPK), activator protein 1 (AP-1), and high-mobility-group-box 1 (HMGB1) protein was done.

Only the person responsible for treatment administration knew the classification of the groups, while a key in the form of numbers was used during the measurement of the samples (blind measurement).

### Biochemical analysis

We kept the clotted blood samples at room temperature for 15 min to half an hour. They were centrifuged at 3,000 × *g*, 4 °C for 10 min, and then their supernatant was subjected to spectrophotometric & ELISA measurements.

BUN was estimated by the rat blood urea nitrogen (BUN) ELISA kit (Catalog # MBS 2611086; MyBioSource, San Diego, CA, USA). The colorimetric test of Reitman & Frankel (1957) was used to measure the levels of serum ALT and AST activity using the Alanine Transaminase Activity Assay Kit (ab105134) and Aspartate Aminotransferase Activity Assay Kit (ab105135), respectively, obtained from Abcam, Cambridge, UK. Serum creatinine and urea levels were measured by the QuantiChrom™ Creatinine Assay Kit, and QuantiChromTM Urea Assay Kit (DIUR-500) obtained from BioAssay Systems Hayward, CA, USA. Serum IL-10 and TNF-α were measured by ELISA using Rat IL-10 ELISA Kit (Catalog #MBS034393; MyBioSource, San Diego, CA, USA) and RayBio® Rat TNF-α ELISA kit (Catalog#: ELR-TNFα; Ray Biotech, Peachtree Corners, GA, USA), respectively.

We removed the liver tissue quickly and rinsed it in ice-cold saline; samples were collected from the right lateral lobe (25 mg tissue was cut and weighed, then homogenized to be used for all the biochemical measurements on the liver by ELISA, Real-time PCR analysis and western blot analysis). Then each sample was divided into three parts. The first part was homogenized using the Mixer Mill MM400 (Retsch, Haan, Germany) in phosphate buffer (pH 6–7). Tissue homogenates were centrifuged at 10,000×*g*, 4° C for 15 min, and its supernatant was used to measure iNOS (Rat iNOS ELISA Kit, Catalog #MBS023874; MyBioSource, San Diego, CA, USA) and Arg-1 (Rat Arginase-1 ELISA Kit, Catalog #MBS762525; MyBioSource, San Diego, CA, USA). The second part was stored in RNA later solution (RNA stabilizing reagent) (Qiagen, Hilden, Germany) at 10 µL per 1 mg of tissue and kept at −80 °C for RNA extraction. The third part was homogenized in RIPA lysis buffer, obtained from Bio Basic Inc. (Markham, ON, Canada) for protein extraction and western blotting analysis.

### Real-time PCR analysis for expression of mRNA of liver c-fos, c-Jun, and high-mobility-group-box1 (HMGB1)

#### Total RNA extraction and reverse transcription

A total of 100 NG of liver tissue was cut and weighed from each stored liver biopsy, then homogenized using the Mixer Mill MM400 (Retsch, Haan, Germany). As the manufacturer’s protocol mentioned, we extracted total RNA using the RNeasy Mini Kit (Qiagen, Hilden, Germany), and used a NanoDrop One spectrophotometer (Thermo Fisher Scientific, Waltham, MA, USA) to determine the RNA concentration and purity, and measured the absorbance at 260 and 280 nm. Pure RNA possesses an A260/A280 ratio of 1.8–2.1. Reverse transcription of RNA (1 µg) was done by the T100 Thermal Cycler (BioRad, Hercules, CA, USA) and the Maxima First Strand cDNA Synthesis Kit (Thermo Fisher Scientific, Waltham, MA, USA), also according to the protocol of the manufacturer.

#### Quantitative real-time PCR

Real-time PCR was done following the protocol of the manufacturer, using the Maxima SYBR Green/ROX qPCR Master Mix (Thermo Fisher, Waltham, MA, USA) by the Step One Plus Real-Time PCR System (Life Technologies, Carlsbad, CA, USA). Primer sequences were as follows:

***-*** *Fos (NM_022197.2)*

Fos Forward AGCATGGGCTCCCCTGTCA

Fos Reverse GAGACCAGAGTGGGCTGCA

*-Jun (NM_021835.3)*

Jun Forward CGACCCCCACTCAGTTCTTGT

Jun Reverse GCAGCGTATTCTGGCTATGCA

*-HMGB1(NM_012963.2)*

HMGB1 Forward ATATGGCAAAAGCGGACAAG

HMGB1 Reverse AGGCCAGGATGTTCTCCTTT

*-β-actin (housekeeping gene, NM_031144.3)*

β-actin Forward CTACCTCATGAAGATCCTCACC

β-actin Reverse AGTTGAAGGTAGTTTCGTGGAT

PCR primers were designed with Gene Runner Software (Hasting Software, Inc., Hasting, NY, USA) from RNA sequences from GenBank. Amplification conditions were 2 min at 50 °C, 10 min at 95 °C and 40 cycles of denaturation for 15 s and annealing/extension at 60 °C for 10 min.

After correction by β-actin expression, the determination of the mRNA expression of each sample was done. The relative expression was calculated using the 2^−∆∆CT^ method.

### Detection of MAPK and AP-1 by western blot technique

Briefly, proteins were extracted from tissue homogenates using ice-cold radioimmunoprecipitation assay (RIPA) buffer supplemented with phosphatase and protease inhibitors (50 mmol/L sodium vanadate, 0.5 mM phenylmethylsulphonyl fluoride, 2 mg/mL aprotinin, and 0.5 mg/mL leupeptin), then centrifugation at 12,000 rpm for 20 min. The protein concentration for each sample was determined using the Bradford assay. Equal amounts of protein (20–30 µg of total protein) were separated by SDS/polyacrylamide gel electrophoresis (10% acrylamide gel) using a Bio-Rad Mini-Protein II system. The protein was transferred to polyvinylidene difluoride membranes (Pierce, Rockford, IL, USA) with a Bio-Rad Trans-Blot system. After transfer, the membranes were washed with PBS and were blocked for 1 h at room temperature with 5% (w/v) skimmed milk powder in PBS. The manufacturer’s instructions were followed for the primary antibody reactions. Following blocking, the blots were developed using antibodies for MAPK, and AP-1 and β-actin supplied by (Thermo Scientific, Waltham, MA, USA, with dilution 1:1,000), incubated overnight at pH 7.6 at 4 °C with gentle shaking. After washing, peroxidase-labeled secondary antibodies (Rockland Immunochemicals, Gilbertsville, PA, USA; with dilution 1:3,000) were added, and the membranes were incubated at 37 °C for 1 h. The ChemiDoc imaging system analyzed band intensity with Image Lab software version 5.1 (Bio-Rad Laboratories Inc., Hercules, CA, USA). The results were expressed as arbitrary units after normalization for β-actin protein expression.

### Statistical analysis

Data was statistically analyzed using GraphPad v.9.0.0 software (GraphPad, San Diego, CA, USA), and the results were expressed as mean ± standard deviation (mean ± SD). The normality of distribution was evaluated by Shapiro-Wilk’s test. Analysis of variance (ANOVA) with the Bonferroni *post hoc* test was done to compare quantitative variables among the groups. Pearson correlation coefficient was used to assess the linear association between quantitative variables. Results were considered statistically significant at *P*-value ≤ 0.05 (Chan, 2003).

## Results

### Serum measurements

The present study showed a significant (*P*-value ≤ 0.05) increase in serum creatinine, BUN, ALT, AST, TNF-α, and IL-10 in the AKI group contrasted with the control group.

Fluid therapy decreased significantly (*P*-value ≤ 0.05) serum creatinine, BUN, ALT, AST, and TNF-α compared to the AKI group. However, values were still significantly (*P*-value ≤ 0.05) higher than the control group. IL-10 showed a minimal reduction compared to the AKI group, yet it increased significantly (*P*-value ≤ 0.05) compared to the control group. Moreover, vildagliptin therapy produced a significant (*P*-value ≤ 0.05) reduction in serum creatinine, BUN, ALT, AST, TNF-α, and IL-10 compared to the AKI group. However, these parameters increased significantly (*P*-value ≤ 0.05) compared to the control group. Serum creatinine showed a more significant (*P*-value ≤ 0.05) reduction in the saline-treated *vs* the vildagliptin-treated group. However, serum TNF-α significantly (*P*-value ≤ 0.05) decreased in the vildagliptin-treated group compared to the saline-treated group (Table 1).

**Table 1:** Serum measurements in the studied groups.

	Control	AKI	AKI+Saline	AKI+Vildagliptin	AKI+Saline+Vildagliptin
**Creatinine (mg\dl)**	0.165 ± 0.02	1.63 ± 0.21*	0.368 ± 0.04*^#^	0.626 ± 0.08*^#^^@^	0.3 ± 0.04^#^^$^
**BUN (mg\dl)**	32.5 ± 2.74	94.33 ± 2.16*	44.83 ± 3.43*^#^	50.67 ± 4.41*^#^	41 ± 5.09*^#^^$^
**ALT (U/L)**	10.72 ± 1.43	40.45 ± 2.84*	23.23 ± 2.03*^#^	25.63 ± 0.88*^#^	19.14 ± 1.18*^#^^@^^$^
**AST(U/L)**	19.5 ± 1.28	57.83 ± 6.64*	33.5 ± 1.38*^#^	31.93 ± 0.91*^#^	23.82 ± 1.48^#^^@^^$^
**TNF-α (PG/ML)**	14.96 ± 1.08	117.3 ± 5.04*	51.83 ± 4.78*^#^	25.89 ± 2.88*^#^^@^	24.45 ± 2.27^#^^@^
**IL-10 (PG/ML)**	111.18 ± 3.8	245.28 ± 10.35*	228.95 ± 7.29*	216.75 ± 6.55*^#^	211.65 ± 18.53*^#^

**Notes:**

* Significant compared to control at *P*-value ≤ 0.05.

# Significant compared to AKI at *P*-value ≤ 0.05.

@ Significant compared to AKI+Saline at *P*-value ≤ 0.05.

$ Significant compared to AKI+Vildagliptin at *P*-value ≤ 0.05.

AKI, Acute Kidney Injury; BUN, Blood urea nitrogen; ALT, alanine transaminase; AST, aspartate aminotransferase; TNF-α, Tumor necrotic factor-α; IL-10, interleukin-10.

Simultaneous treatment by saline and vildagliptin resulted in a significant (*P*-value ≤ 0.05) reduction of serum creatinine, BUN, ALT, AST, TNF-α, and IL-10 compared to the AKI group. It normalized creatinine, ALT, and AST (insignificant difference compared to the control group) (Table 1).

### Tissue markers measured in the liver

**Figure 1:**
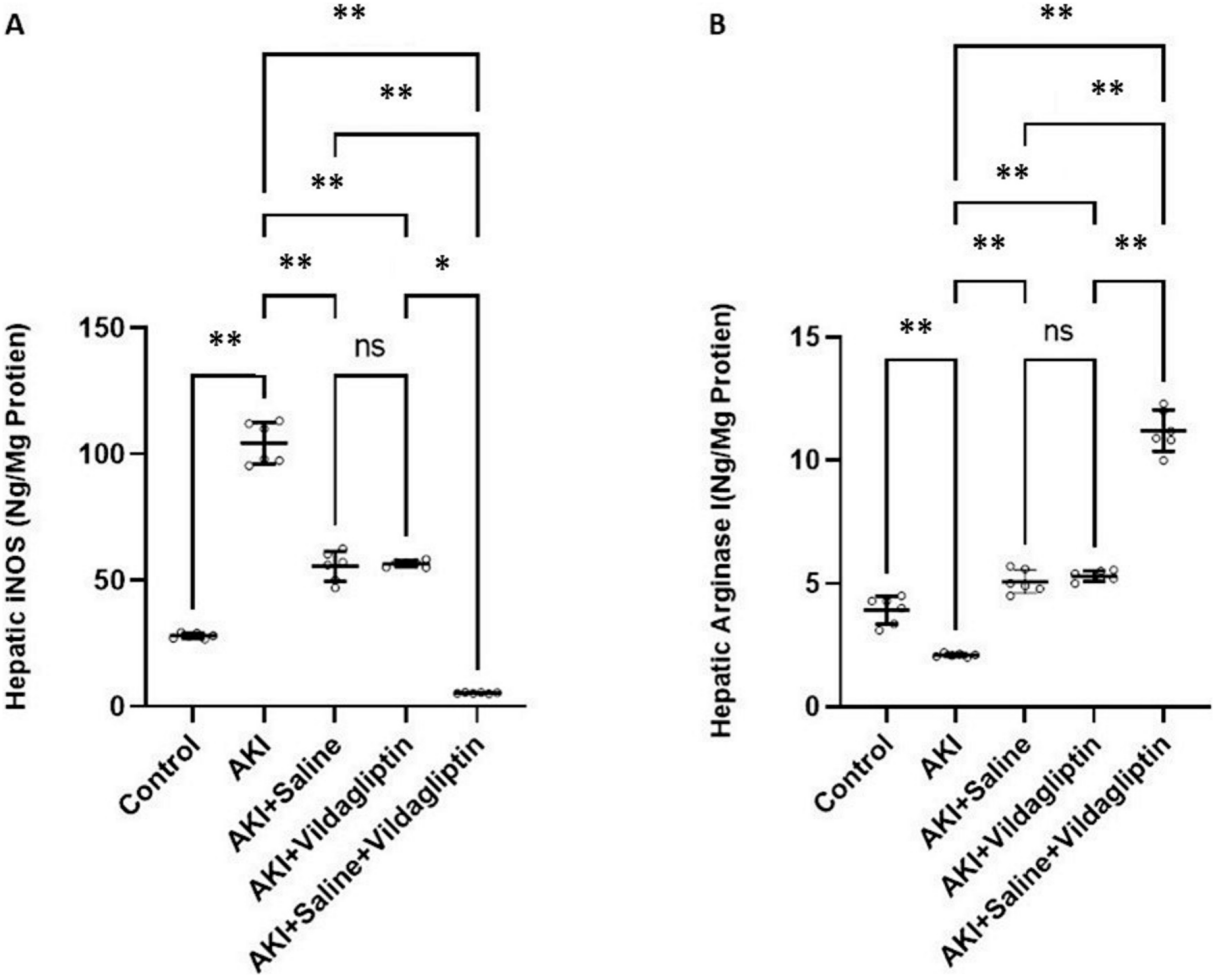
Comparison between hepatic iNOS and arginase I expression levels in the studied groups. Acute Kidney Injury (AKI) in rats was induced by intramuscular (i.m) injection of 50% glycerol (5 ml/kg). Rats were then divided into four groups (*n* = 6 rats/group): untreated AKI, *AKI+saline* (received 1.5 ml of normal saline subcutaneous injection), *AKI+vildagliptin* (treated with oral vildagliptin 10 mg/kg,) and *AKI+saline+vildagliptin:* received saline and vildagliptin. (A) iNOS levels in the AKI group increased significantly (**P-value ≤ 0.01) compared to the control group. Saline, vildagliptin therapy, and combined saline and vildagliptin in AKI significantly (**P-value ≤ 0.01) reduced iNOS levels relative to the untreated AKI group, with an insignificant difference in iNOS levels between saline-treated compared to vildagliptin treated groups. Simultaneous administration of saline and vildagliptin in AKI caused a significant (*P-value ≤ 0.05) reduction in iNOS expression in the AKI group compared to the administration of vildagliptin alone. (B) Arginase I level significantly (**P-value ≤ 0.01) decreased in the AKI group compared to the control group. Saline or vildagliptin therapy significantly (**P-value ≤ 0.01) elevated the levels of arginase I compared to untreated AKI. Simultaneous administration of both saline and vildagliptin raised the arginase I level significantly (**P-value ≤ 0.01) compared to the AKI group, saline-treated group, and vildagliptin-treated group. Data were analyzed with one-way ANOVA with Bonferroni *post-hoc* test. Data are expressed as mean ± SD.

iNOS levels in the AKI group increased significantly (*P*-value ≤ 0.01) compared to the control group. Saline, vildagliptin therapy, and combined saline and vildagliptin in AKI significantly (*P*-value ≤ 0.01) reduced iNOS levels relative to the untreated AKI group, with an insignificant difference in iNOS levels between saline-treated compared to the vildagliptin treated groups. Simultaneous administration of saline and vildagliptin in AKI caused a significant (*P*-value ≤ 0.05) reduction in iNOS expression in the AKI group compared to the administration of vildagliptin alone (Fig. 1A).

Arg-1 level significantly (*P*-value ≤ 0.01) decreased in the AKI group compared to the control group. Saline or vildagliptin therapy significantly (*P*-value ≤ 0.01) elevated the levels of Arg-1 compared to the untreated AKI. Simultaneous administration of both saline and vildagliptin raised Arg-1 level significantly (*P*-value ≤ 0.01) compared to the AKI group, the saline-treated group, and the vildagliptin-treated group (Fig. 1B).

In terms of quantitative comparison among the studied groups: iNOS levels increased by 372% in the AKI group relative to the control group. Saline therapy reduced it by 53.21% relative to the levels of the AKI group. Vildagliptin therapy reduced it by 54.241% relative to the AKI group. Combined saline and vildagliptin reduced it by 45.27% relative to the levels of the AKI group. Arginase I levels declined by 53.43% in the AKI group relative to the control group. Saline therapy increased it by 241.9% relative to the AKI group. Vildagliptin therapy increased it by 252.8% relative to the AKI group. Combined saline and vildagliptin increased it by 533.8% relative to the AKI group’s levels.

Evaluation of the relative expression of genes of c-fos, c-Jun, and HMGB1 by PCR revealed a significant (*P*-value ≤ 0.05) increase in the relative expression of c-fos, c-Jun, and HMGB1 in the AKI group relative to the control group. Saline or vildagliptin treatment significantly (*P*-value ≤ 0.05) decreased the relative expression of these signaling molecules compared to the AKI group, but values remained significantly (*P*-value ≤ 0.05) increased in comparison with the control group. There was no significant (*P*-value ≤ 0.05) difference in these markers’ values in the saline-treated *vs* vildagliptin-treated group. Cotreatment with saline and vildagliptin caused a more significant (*P*-value ≤ 0.05) reduction of these signaling molecules’ expression than the AKI group. However, values were still significantly higher than (*P*-value ≤ 0.05) those of the control group (Table 2).

**Table 2:** Tissue markers measured in the liver of the studied groups by Real-time PCR analysis.

	Control	AKI	AKI+Saline	AKI+Vildagliptin	AKI+Saline+Vildagliptin
**cFOS** **(Relative Expression)**	1.013 ± 0.025	4.76 ± 0.332*	2.801 ± 0.304*^#^	2.583 ± 0.348*^#^	1.936 ± 0.098*^#^^@^^$^
**cJUN** **(Relative Expression)**	1.028 ± 0.031	6.03 ± 0.135*	3.016 ± 0.171*^#^	3.11 ± 0.357*^#^	2.185 ± 0.303*^#^^@^^$^
**HMGB1** **(Relative Expression)**	1.07 ± 0.04	5.53 ± 0.17*	2.42 ± 0.275*^#^	2.58 ± 0.35*^#^	2.02 ± 0.084*^#^^@^^$^

**Notes:**

* Significant compared to control at *P*-value ≤ 0.05.

# Significant compared to AKI at *P*-value ≤ 0.05.

@ Significant compared to AKI+Saline at *P*-value ≤ 0.05.

$ Significant compared to AKI+Vildagliptin at *P*-value ≤ 0.05.

AKI, Acute Kidney Injury; TNF-α, Tumor necrotic factor-α; IL-6, interleukin-6; HMGB, High-mobility-group-box protein.

**Figure 2:**
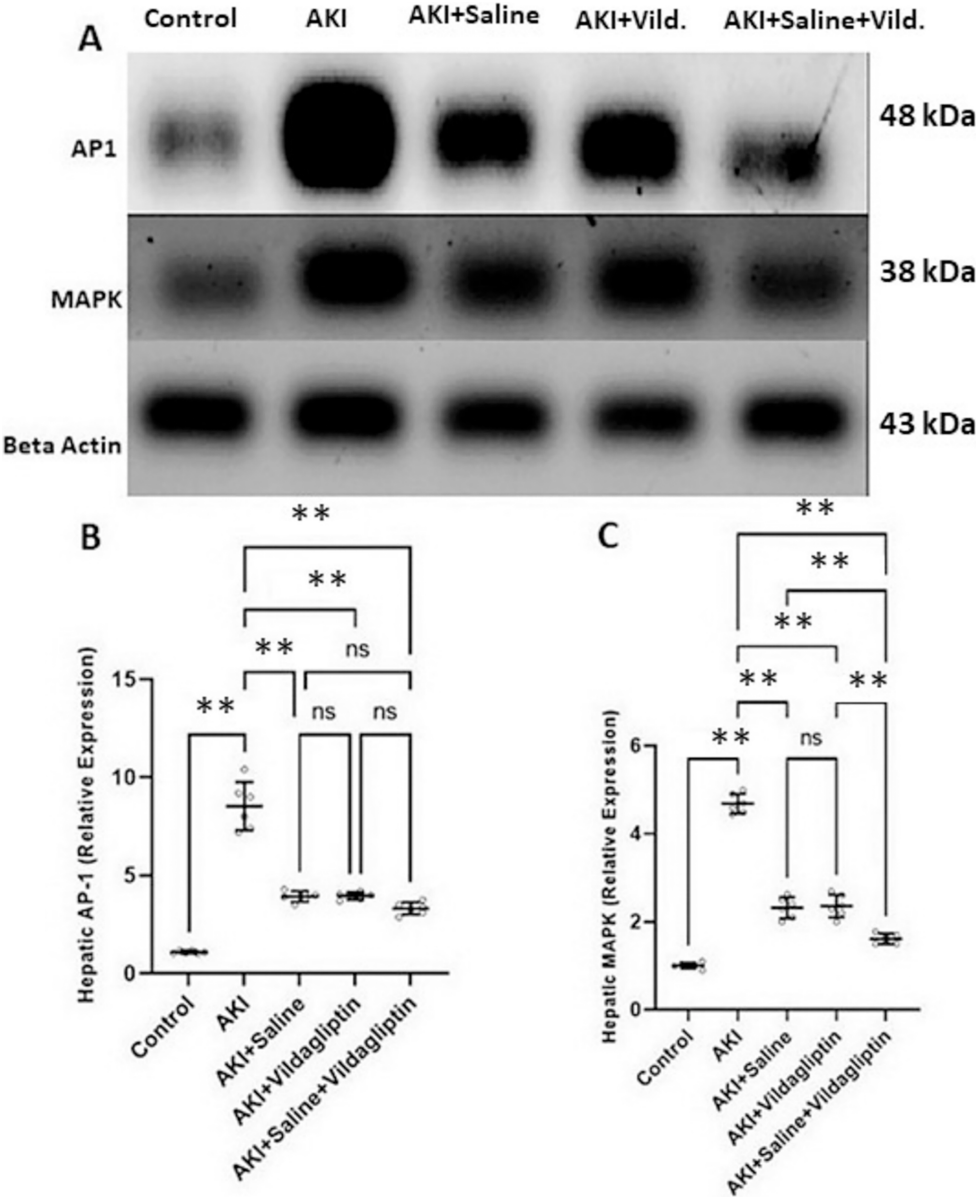
Western blot analysis of hepatic expression AP1 and MAPK in the studied groups. Acute kidney injury (AKI) in rats was induced by intramuscular (im) injection of 50% glycerol (5 ml/kg). Rats were then divided into four groups (*n* = 6 rats/group): untreated AKI, AKI+saline (received 1.5 mL of normal saline subcutaneous injection), AKI+vildagliptin (treated with oral vildagliptin 10 mg/kg), and AKI+saline+vildagliptin: received saline and vildagliptin. (A) Cropped images of the WB gel images of hepatic AP1 and MAPK relative to β-actin. (B) A highly significant (**P-value ≤ 0.01) increase in the expression of AP-1 in the AKI group relative to the control group. Saline, vildagliptin, and combined treatment significantly (**P-value ≤ 0.01) decreased AP-1 expression compared to the AKI group. There was no significant difference in AP-1 expression between the three treated groups. (C) A highly significant (**P-value ≤ 0.01) increase in the expression of MAPK in the AKI group relative to the control group. Saline, vildagliptin, and combined treatment significantly (**P-value ≤ 0.01) decreased MAPK expression compared to the AKI group. There was no significant difference in MAPK expression between the saline and vildagliptin-treated groups. However, in the combined saline+vildagliptin, MAPK expression was significantly (**P-value ≤ 0.01) decreased compared to the saline and vildagliptin-treated groups. Data were analyzed with one-way ANOVA with Bonferroni *post-hoc* test. Data are expressed as mean ± SD.

Evaluation of AP-1 by WB revealed a highly significant (*P*-value ≤ 0.01) increase in the expression of AP-1 in the AKI group relative to the control group. Saline, vildagliptin, and combined treatment significantly (*P*-value ≤ 0.01) decreased AP-1 expression compared to the AKI group. There was no significant difference in AP-1 expression between the three treated groups (Figs. 2A and 2B).

Assessing MAPK expression revealed a highly significant (*P*-value ≤ 0.01) increase in the AKI group relative to the control group. Saline, vildagliptin, and combined treatment significantly (*P*-value ≤ 0.01) decreased MAPK expression compared to the AKI group. There was no significant difference in MAPK expression between the saline and the vildagliptin-treated groups. However, in the combined saline+vildagliptin-treated group, MAPK expression significantly (*P*-value ≤ 0.01) decreased compared to the saline-treated group and the vildagliptin-treated group (Figs. 2A and 2C).

Moreover, significant (*P* value ≤ 0.01) positive correlations were found between hepatic expression of iNOS and c-fos, c-Jun, MAPK, AP-1, and HMGB1 expression (r = 0.966, 0.980, 0.977, 0.987, 0.979 respectively), and significant (*P* value ≤ 0.05) negative correlations between hepatic expression of Arg-1 and c-fos, c-Jun, MAPK, AP-1 and HMGB expression (r = −0.450, −0.461, −0.513, −0.383, −0.461 respectively).

## Discussion

AKI produces well-known side effects that mostly lead to a high death rate, like accumulation of uremic toxins, metabolic acidosis, disturbances in electrolyte levels, and fluid overload. However, a considerable percentage of AKI-related mortality is not a result of kidney function loss alone or AKI treatment complications. Instead, the increased risk of poor outcomes for these patients may be explained by the affection of other organs in AKI, including the liver, in a process called ‘organ crosstalk’ (Doi & Rabb, 2016). A growing body of data suggests that AKI-induced distant organ crosstalk necessitates a disturbance of the immunological balance and the production of inflammatory mediators (Lane et al., 2013).

AKI causes the induction of oxidative stress and triggers apoptosis, inflammatory mediators, and tissue damage in the liver (Lee et al., 2018). These findings follow the present study’s results, as we demonstrated a remarkable increase in the ALT and AST in the AKI group relative to the control group. The beneficial effect of saline injection & vildagliptin treatment in improving kidney functions was quite clear. It significantly lowered the AST & ALT levels in both treated groups compared to the untreated AKI group, confirming previous studies correlating AKI with hepatic dysfunction (Fadillioglu et al., 2008; Park et al., 2012).

The present results showed a significant decrease in serum IL-10 and TNF-α levels with the administration of vildagliptin compared to the untreated AKI group. The anti-inflammatory effect of vildagliptin by reducing tissue TNF-α levels was demonstrated in other models (He et al., 2017; El Batsh et al., 2015). DPP-4i could protect against organ injuries by reducing oxidative stress, dysfunction of mitochondria (Bayrami et al., 2018), and the apoptotic executive caspase “caspase-3” protein expression (El-Marasy, Abdel-Rahman & Abd-Elsalam, 2018).

Following our results, Kern et al. (2012) reported that the DPP-4i linagliptin alleviated liver steatosis and inflammation by minimizing hepatic infiltration by macrophages and decreasing lobular inflammation and TNF-α relative expression. Moreover, Jiang et al. (2018) demonstrated that sitagliptin decreased IL-1β, IL-6, and TNF-α levels in a dose-dependent manner. The antioxidant and anti-inflammatory effects of the DPP-4i vildagliptin shown in the current work may be caused by the diminished infiltration of local M1 macrophage and mast cells (MCs), which decreased TNF-α, IL-6, and TGF-β1 in their secretory granules (He et al., 2017).

The exact mechanisms which lead to the pathological changes in the distant organs with AKI are still unclear. Despite the elevation of proinflammatory cytokines in the kidney and these distant organs following AKI, their origins are still unknown (Shang et al., 2020). Macrophages critically impact liver damage and healing pathogenesis in acute and chronic hepatic diseases. Based on the distinct phenotypes and origins, hepatic macrophages (Kupffer cells KCs) can clear pathogens and promote/or inhibit liver inflammation (Sun et al., 2017).

Macrophage polarization reflects the functional differences between macrophages. Classically activated M1 macrophages produce inflammatory mediators, whereas alternatively activated M2 macrophages produce anti-inflammatory cytokines (Qian et al., 2019). M1 macrophages express iNOS, which metabolizes arginine to NO and citrulline. Further downstream reactive nitrogen species may be formed by NO metabolism, while the citrulline–NO cycle can synthesize NO from citrulline (Sica, Invernizzi & Mantovani, 2014); in other words, iNOS is considered a hallmark molecule of M1 macrophages (Sica & Mantovani, 2012).

Based on previous information, our results revealed M1 macrophage polarization in injured liver tissue, evidenced by the significant elevation of iNOS level in the AKI group compared to the control group. This increased level was probably a cause of liver injury secondary to AKI. Many studies showed that M1 KCs could produce proinflammatory factors such as TNF-α, interleukins, ROS, and the iNOS itself, which can induce liver damage (Dey, Allen & Hankey-Giblin, 2015). Moreover, it is worth noting that TNF-α can activate M1 KCs themselves (Shang et al., 2020).

For investigation of other mechanisms which may participate in hepatic M1 macrophage polarization of the AKI group in our current study, the relative expression of several signaling molecules was measured in the liver tissue. The control of iNOS expression occurs mainly at the transcriptional level, and the human and murine iNOS promoters contain several binding sites for AP-1. This major transcription factor has a role in iNOS gene transcription (Lee et al., 2009).

AP-1 comprises proteins that belong to the immediate-early genes; Jun and fos (Chung, Lee & Kim, 2004). Hasselblatt et al. (2007) claimed that the lack of c-Jun in lipopolysaccharide-mediated hepatitis markedly reduced the induction of iNOS RNA. This finding may explain the significant positive correlation between iNOS content and the expression of AP-1, c-Jun, and c-fos in our present study. c-Jun N-terminal kinase (JNK) is substantial for cytokine-induced cell death. The transcription factor c-Jun/AP-1, a prototypic target of JNK, is highly expressed in the liver of patients with acute hepatic injury (Tiegs, Hentschel & Wendel, 1992). The binding of TNF-α to its receptors causes activation of JNK, nuclear factor B (NF-B), and AP-1 (Maeda et al., 2003). This pathway provides an interpretation for our results that revealed a significant elevation in the relative expression of c-Jun, AP-1, and c-fos in the AKI group relative to the control group.

Furthermore, activated MAPK increases by inflammatory conditions *via* activating the transcription of the iNOS gene (Chan & Riches, 2001); our results verified this mechanism and demonstrated a significant positive correlation between the hepatic content of iNOS and the relative expression of MAPK. It also explains the considerable rise in the expression of MAPK in the AKI group compared to the control group. It is worth noting that there is also an interrelation between MAPK and AP-1. MAPK signaling pathways influence the activity of AP-1 by boosting its protein transcription and phosphorylation (Suh et al., 2008).

HMGB is a non-histone DNA-binding nuclear protein that participates in nucleosome stabilization. Inhibiting extracellular HMGB reduces inflammation and protects against various inflammatory liver disorders (Yokoi et al., 2018). Moreover, HMGB reportedly acts as an endogenous damage-associated molecular pattern (DAMPs) after their extracellular release by necrotic cells or their active secretion by macrophages, which leads to the induction and magnification of inflammatory processes (Mihm, 2018). HMGB has a substantially longer therapy window for patients and contributes remarkably to multiple organ damage (Zhao et al., 2018).

Wen et al. (2020) detected translocation of HMGB in hepatocytes after intestinal ischemia/reperfusion (I/R) challenge, increased M1 macrophages, which accompanied HMGB release in the liver, and an increased colocalization of HMGB and anti-HMGB treatment alleviated these effects. Their results revealed that HMGB neutralization and inhibition were associated with liver injury attenuation. This report is concomitant with our findings that demonstrated a significant rise in the hepatic relative expression of HMGB in the AKI group relative to the control group, besides the significant positive correlation between the relative expression of both HMGB and iNOS.

Opposite to M1 macrophages, M2 macrophages exhibit anti-inflammatory activity, promote damage repair (Shi et al., 2018), and express arginase enzyme, which acts on arginine, hydrolyzing it into ornithine and urea. Arg-1 is considered an M2 macrophage marker (Heo et al., 2014). Therefore, it is possible to conclude that M2 polarization in liver tissue in our treated groups is evidenced by the significant elevation of Arg-1 relative expression in these groups compared to the AKI group and even to the control group. In our study, this M2 polarization is possibly the cause of improved liver tissue in the treated groups, as clearly observed in the form of a significant decline in ALT and AST serum levels in the treated groups compared to the AKI group.

Macrophages express DPP-4 as a crucial factor in immunity and inflammation programs (Zhuge et al., 2016). Zhuge et al. (2016) clarified that DPP-4i regulates macrophage polarization and its consequent inflammatory inhibition of NAFLD progression. Similarly, saxagliptin enhanced M1 to M2 macrophage polarization, as evidenced by reduced M1 marker and elevated M2 marker *in vivo* and *in vitro* (Yang et al., 2018).

Another study by Nishida et al. (2020) examined the influence of DPP-4i (linagliptin) on *in vitro* and *in vivo* macrophage polarization. It showed that IFN-γ treatment reduced M2 markers’ levels, while treatment with linagliptin avoided these reductions. All previous studies align with our results that revealed a significant increase in the hepatic content of Arg-1 (indicating M2 polarization) and a significant decline in the liver content of iNOS after vildagliptin administration. However, the work done by Brenner et al. (2015) partially contradicts the previous results, as they revealed that DPP-4i did not affect the number of M1 macrophages but only increased M2 macrophages in atherosclerotic aorta dissected from mice.

Furthermore, Yang et al. (2018) demonstrated that saxagliptin reduced lipid accumulation and alleviated hepatic inflammation by minimizing inflammation in diabetic rats. They clarified that the anti-inflammatory impact of saxagliptin promotes M2 macrophage polarization and suppresses TNF-α expression in the M1 macrophage. Another *in-vitro* study by Zhuge et al. (2016) concluded that M1 polarization and augmented ROS production in cultured peritoneal macrophages markedly decreased in the presence of linagliptin with increased expression of M2 macrophage markers. These studies partly interpret why vildagliptin therapy was associated with hepatic protection against injury secondary to AKI. The strong assumption that vildagliptin is the cause of this significant decrease in TNF-α level is supported by the insignificant difference between the AKI+vildagliptin group and the combined AKI+saline+vildagliptin group. So, the effect of saline on TNF-α is possibly ignorable.

The binding of TNF-α to its receptors activates JNK, NF-kB, and AP-1 (Maeda et al., 2003), concluding that the significant decrease in serum TNF-α level would suppress these changes. This effect possibly explains the significant reduction in expression of AP-1, c-Jun, and c-fos (as a part of AP-1) in the AKI+vildagliptin group compared to the AKI group.

Guo et al. (2020) indicated that alogliptin treatment reduced IL-1β-induced production of ROS and release of TNF-α and IL-6. Additionally, they observed that alogliptin inhibited JNK/AP-1 signaling by decreasing IL-1β-induced phosphorylation of JNK and c-Jun and c-fos expression. This study agrees with our results, which revealed a significant decrease in MAPK and c-Jun relative expression in the AKI+vildagliptin group relative to the AKI group. Putting all previous interrelations together, in addition to the conclusion of Zhuge et al. (2016), which mentioned that treatment with linagliptin elevated the expression of M2 macrophage markers (including Arg-1), enables us to explain the significant negative correlation between the expression of hepatic Arg-1 and the expression of MAPK, AP-1, c-Jun and c-fos demonstrated by our study.

Wang et al. (2017) also demonstrated that DPP-4i reduced the accumulation of inflammatory macrophages and motivated alternative M2 macrophage polarization. However, they proposed a different explanation suggesting that this effect of DPP-4i on the regulation of macrophages is dependent on reduced apoptosis and oxidative stress rather than decreased inflammation. Other studies have demonstrated that arginase activation may trigger apoptosis of iNOS-expressing cells. A mechanism that is possibly involved in these apoptotic effects may be that, after combined activation of arginase and iNOS, the consequent competition for their shared substrate L-arginine results in reduced arginine availability and a shift of iNOS function towards proapoptotic characteristics (Bronte et al., 2003).

## Conclusions and recommendations

**Figure 3:**
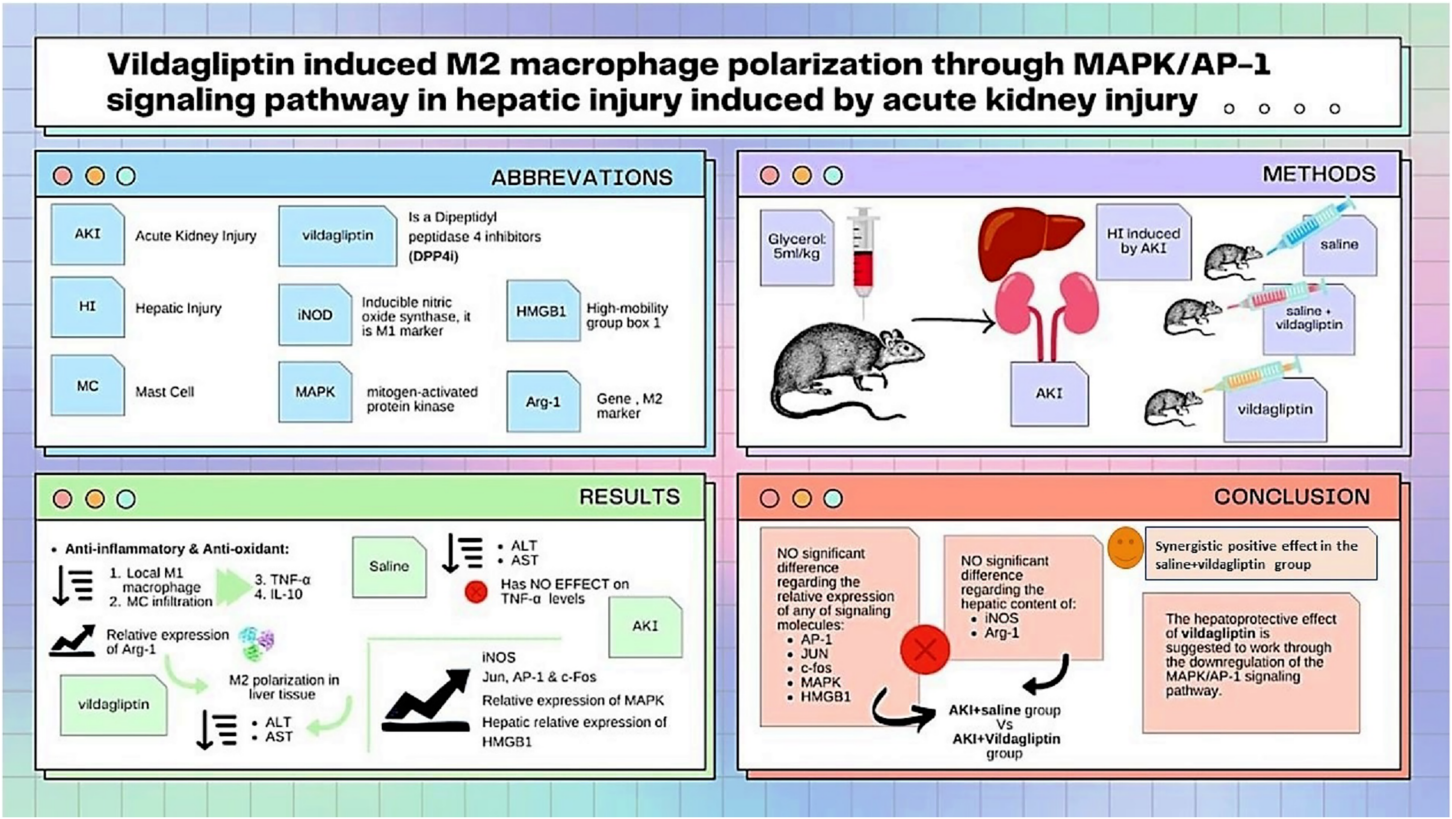
Illustration summarizes the study design, methods, and major findings. Acute kidney injury (AKI) in rats was induced by intramuscular (i.m) injection of 50% glycerol (5 ml/kg). Rats were then divided into four groups (*n* = 6 rats/group): untreated AKI, AKI+saline( received 1.5 mL of normal saline subcutaneous injection), AKI+vildagliptin (treated with oral vildagliptin 10 mg/kg), and AKI+saline+vildagliptin: received saline and vildagliptin. The study compared the protective effect of saline and vildagliptin DPP-4 inhibitor. There was an insignificant difference regarding the relative expression of AP-1, JUN, c-fos, MAPK, and HMGB1between the AKI+saline group and AKI+vildagliptin group. Even the hepatic content of the M1 marker (iNOS) and M2 marker (Arg-1) showed insignificant differences between both groups. However, combined therapy produced more pronounced changes in these markers, as the difference in their relative expression between the AKI+saline+vildagliptin group and both the AKI+saline group and AKI+vildagliptin group was significant. We suggest that the vildagliptin hepatoprotective effect involves the downregulation of the MAPK/AP-1 signaling pathway.

As summarized in Fig. 3, our study compared the protective effect of saline and the DPP-4i vildagliptin. Regarding the previously mentioned signaling molecules (AP-1, c-Jun, c-fos, MAPK, and HMGB1), there was an insignificant difference in the relative expression of all of them between the AKI+saline group and AKI+vildagliptin group. The hepatic content of the M1 marker (iNOS) and M2 marker (Arg-1) showed insignificant differences between both groups. However, combined therapy produced more pronounced changes in these markers, as the difference in their relative expression between the AKI+saline+vildagliptin group and both the AKI+saline group and the AKI+vildagliptin group was significant. We conclude that the combined fluid and vildagliptin therapy showed a superior outcome compared to either treatment alone. A possible mechanism for this synergistic action of the combined therapy may involve the downregulation of the MAPK/AP-1 signaling pathway.

Further research is needed to assess the liver and kidney tissues in AKI and the effect of therapy on tissue morphology and macrophage CD markers using different histopathological and ultrastructural techniques.

## Supplemental Information

10.7717/peerj.14724/supp-1 Supplemental Information 1 AKI significantly increased AP-1 protein expression while administering Saline, vildagliptin, and combined treatment significantly downregulated AP-1 expression compared to the AKI group.

10.7717/peerj.14724/supp-2 Supplemental Information 2 Beta actin as a western blotting loading control.

10.7717/peerj.14724/supp-3 Supplemental Information 3 AKI significantly increased MAPK protein expression while administering Saline, vildagliptin, and combined treatment significantly downregulated MAPK expression compared to the AKI group. Combined treatment significantly downregulated MAPK expression compared to the saline-treated group and the vildagliptin-treated group.

10.7717/peerj.14724/supp-4 Supplemental Information 4 Checklist.

## Additional Information and Declarations

### DNA Deposition

The following information was supplied regarding the deposition of DNA sequences: Sequences are available at GenBank: NM_022197.2, NM_021835.3, NM_012963.2, NM_031144.3.

### Data Availability

The following information was supplied regarding data availability: The data is available at Figshare: Amin, Shaimaa (2022): data files, liver in AKI. figshare. Dataset. https://doi.org/10.6084/m9.figshare.17680328.v1.
