# Supplementary figures and images for: Combined saline and vildagliptin induced M2 macrophage polarization in hepatic injury induced by acute kidney injury
Source: PeerJ. 2023 Feb 13;11:e14724. doi: 10.7717/peerj.14724 (PMC9933746; doi:10.7717/peerj.14724)

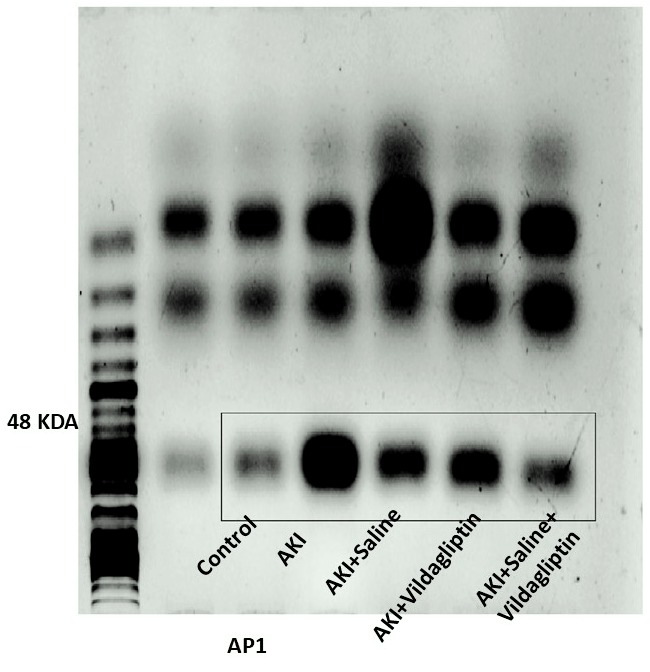

Supplement: Supplemental Information 1 [file peerj-11-14724-s001.jpg]

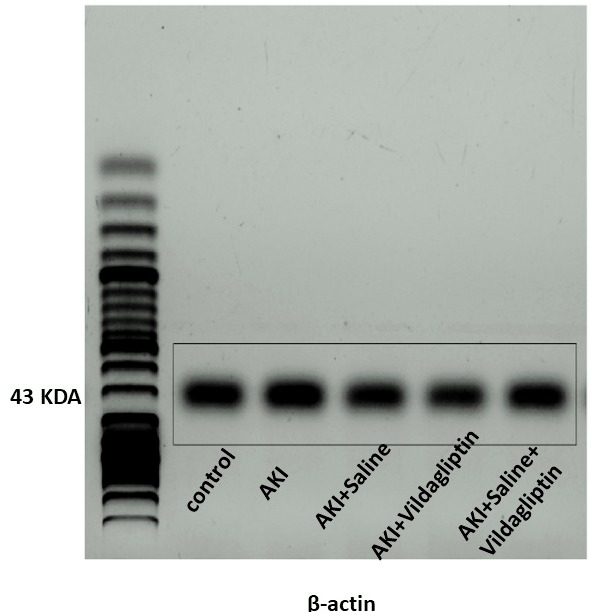

Supplement: Supplemental Information 2 [file peerj-11-14724-s002.jpg]

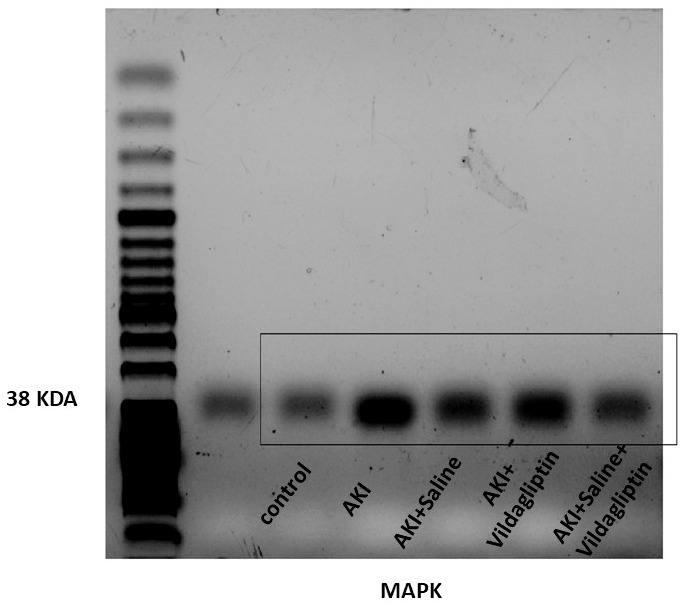

Supplement: Supplemental Information 3 — Combined treatment significantly downregulated MAPK expression compared to the saline-treated group and the vildagliptin-treated group. [file peerj-11-14724-s003.jpg]
